# The Effect of Essential Oils and Bioactive Fractions on *Streptococcus mutans* and *Candida albicans* Biofilms: A Confocal Analysis

**DOI:** 10.1155/2015/871316

**Published:** 2015-03-02

**Authors:** Irlan Almeida Freires, Bruno Bueno-Silva, Lívia Câmara de Carvalho Galvão, Marta Cristina Teixeira Duarte, Adilson Sartoratto, Glyn Mara Figueira, Severino Matias de Alencar, Pedro Luiz Rosalen

**Affiliations:** ^1^Pharmacology, Anesthesiology and Therapeutics, Department of Physiological Sciences, Piracicaba Dental School, University of Campinas, 13414-903 Piracicaba, SP, Brazil; ^2^Department of Microbiology, Institute of Biomedical Sciences, University of São Paulo, 05508-900 São Paulo, SP, Brazil; ^3^Research Center for Chemistry, Biology and Agriculture, University of Campinas, 13083-970 Campinas, SP, Brazil; ^4^Department of Agri-Food Industry, Food and Nutrition, “Luiz de Queiroz” College of Agriculture, University of São Paulo, 13418-900 Piracicaba, SP, Brazil

## Abstract

The essential oils (EO) and bioactive fractions (BF) from *Aloysia gratissima, Baccharis dracunculifolia, Coriandrum sativum, Cyperus articulatus,* and *Lippia sidoides* were proven to have strong antimicrobial activity on planktonic microorganisms; however, little is known about their effects on the morphology or viability of oral biofilms. Previously, we determined the EO/fractions with the best antimicrobial activity against *Streptococcus mutans* and *Candida* spp. In this report, we used a confocal analysis to investigate the effect of these EO and BF on the morphology of *S. mutans* biofilms (thickness, biovolume, and architecture) and on the metabolic viability of *C. albicans* biofilms. The analysis of intact treated *S. mutans* biofilms showed no statistical difference for thickness in all groups compared to the control. However, a significant reduction in the biovolume of extracellular polysaccharides and bacteria was observed for *A. gratissima* and *L. sidoides* groups, indicating that these BF disrupt biofilm integrity and may have created porosity in the biofilm. This phenomenon could potentially result in a weakened structure and affect biofilm dynamics. Finally, *C. sativum* EO drastically affected *C. albicans* viability when compared to the control. These results highlight the promising antimicrobial activity of these plant species and support future translational research on the treatment of dental caries and oral candidiasis.

## 1. Introduction

It has been long known that biofilms formed by* Streptococcus mutans* and* Candida albicans *are implicated in the establishment of dental caries and oral candidiasis, respectively, and constitute complex structures, often composed of interactive microorganisms which are organized for increased metabolic efficiency, resistance to stress, and virulence [1] with a sophisticated* quorum sensing* system [2].

Extracellular polysaccharides (EPS) produced by* S. mutans* glycosyltransferases are the main constituents in the matrix of cariogenic biofilms and are recognized as critical virulence factors associated with dental caries [3]. An EPS-rich matrix creates highly adhesive, interconnected, and acidic environments, which ultimately leads to the clinical onset of cavitation through acid demineralization of the adjacent enamel [4]. The EPS matrix provides a three-dimensional scaffold for biofilm development and assembly, which confers increased resistance to antimicrobials and creates signaling pathways [3].

Biofilm formation on host tissues or medical devices mediated either by biotic (cell-surface proteins) or abiotic (surface hydrophobicity) factors also plays a role in the pathogenesis of yeast infections [5].

Therefore, the development of specific-targeted therapies attenuating microbial virulence is of utmost importance and thus contributing to the management of dental caries and oral candidiasis, which have considerably affected the population worldwide [6, 7]. With this perspective, essential oils (EO) have been shown to be effective antimicrobial agents against a number of gram-positive bacteria and yeasts. They are complex, volatile compounds produced by aromatic plants as secondary metabolites [8], and the presence of complex chemical structures thereof may explain their antimicrobial effects upon biofilms of clinical relevance.

In our previous studies, we screened twenty EO and their bioactive fractions and selected those with the most promising antimicrobial activity against* S. mutans *[9] and* Candida* spp. [10, 11] as follows:* Aloysia gratissima *(fraction Ag_4_),* Baccharis dracunculifolia *(fraction Bd_2_),* Coriandrum sativum *(EO and fraction Cs_4_),* Lippia sidoides *(fraction Ls_3_), and* Cyperus articulatus *(EO). In general, Cs_4_ and Bd_2_ inhibited more than 90% of* in vitro S. mutans* biofilm formation at low concentrations (31.2 *μ*g/mL), followed by Ag_4_ (62.5 *μ*g/mL) and Ls_3_ (125 *μ*g/mL) [9]. The EO from* C. sativum* and* C. articulatus* inhibited* C. albicans* biofilm from 62.5 *μ*g/mL [11] and 1.95 *μ*g/mL [10], respectively. Furthermore, these studies have demonstrated by scanning electron microscopy analysis that the EO disrupt biofilm integrity, but little is known about the effect of these EO on the architectural structure and viability of oral biofilms.

Thus, based on these previous results and on the relevance of discovering new agents for the management of biofilm-dependent diseases, we investigated through a confocal analysis the effect of the EO and bioactive fractions earlier mentioned on the morphology of* S. mutans* biofilms by assessing thickness, biovolume, and architecture and on the metabolic viability of* C. albicans* biofilms.

## 2. Material and Methods

### 2.1. Plant Material

Plant species were obtained from the germoplasm bank of the Collection of Medicinal and Aromatic Plants (CPMA) at the Research Center for Chemistry, Biology, and Agriculture (CPQBA), University of Campinas (UNICAMP), SP, Brazil (available at http://webdrm.cpqba.unicamp.br/cpma/). The plant material was collected between November and January, during the morning. Voucher specimens were deposited in the herbarium of the Institute of Biology at the University of Campinas (Campinas, SP, Brazil) and also registered in the herbarium of CPQBA, receiving identification numbers (Table 1).

### 2.2. Essential Oil Extraction and Fractionation

The EO were obtained through hydrodistillation of the leaves or bulbs for 3 hours in a Clevenger-type system. The aqueous phase was extracted with dichloromethane and the organic layer was then isolated, dried with anhydrous sodium sulphate (Na_2_SO_4_) to remove any trace of water, and filtered. This step is critical to absorb remaining moisture in the organic phase, resulting in water-free oil content. The solvent was finally evaporated to obtain the crude EO [12]. The EO were stored at −20°C in amber glass vials to be subsequently fractionated. Emulsions of the fractions or crude oils were prepared using propylene glycol (v/v) as vehicle before undergoing microbiological testing.

The EO were fractionated using the dry column method (cellulose column 2 cm × 20 cm) with Si gel 60 (Merck, Darmstadt, Germany) as the stationary phase and dichloromethane as the mobile phase, previously chosen by Thin Layer Chromatography. After elution, columns were separated into different parts for each EO based on polarity. The fractions obtained were chemically characterized by Thin Layer Chromatography and Gas Chromatography coupled to Mass Spectrometry (GC-MS) and tested for their antimicrobial activity. The selected EO and bioactive fractions showing the best antibacterial/antifungal effects (previously found by Galvão et al. [9] and Freires et al. [11]) are listed as follows:* A. gratissima *(fraction Ag_4_),* B. dracunculifolia *(fraction Bd_2_),* C. sativum *(fraction Cs_4_ and EO),* C. articulatus *(EO), and* L. sidoides *(fraction Ls_3_).

All chemical wastes generated in this study were treated in accordance with the principles of the Environmental Ethics Board at the University of Campinas, SP, Brazil, under protocol number 324/2009.

### 2.3. Phytochemical Analysis of the Essential Oils and Bioactive Fractions by Gas Chromatography Coupled to Mass Spectrometry (GC-MS)

Volatile constituents were identified using a Hewlett-Packard 6890 gas chromatograph coupled with an HP-5975 mass selective detector and HP-5 capillary column (30 m × 0.25 mm × 0.25 *μ*m diameter). GC-MS analysis was performed using split injection (40 : 1), with the injector set at 220°C, column set at 60°C, with a heating ramp of 3°C/min and a final temperature of 240°C, and the MS detector set at 250°C. Helium was used as a carrier gas at 1 mL/min. The GC-MS electron ionization system was set at 70 eV. Samples of the bioactive fraction or crude oil were solubilized in ethyl acetate for the analysis. Then, retention indices (RIs) were determined by coinjection of hydrocarbon standards (alkanes C_8_–C_30_) and test samples under the same conditions. The oil components were identified by comparison with the data from the NIST 05 library, international literature, and by coinjection of authentic standards [11, 13].

### 2.4. Preparation of* S. mutans* and* C. albicans* Suspensions

Reference strains of* S. mutans* UA 159 (ATCC 700610, serotype* c*) and* C. albicans *CBS 562 were used.

A starter culture of* S. mutans* was prepared using ultrafiltered (10 kDa cutoff membrane; Prep/Scale; Millipore, MA) tryptone yeast extract (UFTYE, pH 7.0) supplemented with 1% (w/v) glucose, incubated at 37°C, 5% CO_2_, overnight. This starter was used to prepare bacterial inoculum at midexponential growth phase for biofilm formation (OD_600 nm_, 2 × 10^6^ CFU/mL) (adapted from da Cunha et al. [14]).

*C. albicans* was grown in yeast nitrogen base (YNB) (Himedia, Mumbai, India) supplemented with 50 mM glucose. Fifty millimeters of medium was inoculated with yeast colonies from Sabouraud Dextrose Agar (Himedia, Mumbai, India) plates, followed by incubation for 24 h at 37°C. Cells were harvested (1200 rpm, 10 minutes, 10°C), washed twice with 0.15 M phosphate-buffered saline (pH 7.2, Ca^2+^- and Mg^2^-free), resuspended in 40 mL of PBS, and read on spectrophotometer to have a concentration of 5 × 10^6^ CFU/mL (530 nm, *λ* = 0.08–0.1) (adapted from Kuhn et al. [15]).

### 2.5. Preparation and Treatment of Biofilms

*S. mutans* biofilms were developed on saliva-coated glass slides (surface area 324 mm^2^) immersed in 12-well flat-bottom cell culture plates (TPP, Trasadingen, Switzerland) in triplicate. Human whole saliva was collected from two donors (Research Ethics Committee, Piracicaba Dental School, University of Campinas, protocol #087/2011), clarified by centrifugation (10000 g, 4°C, 10 min), sterilized and diluted (1 : 1) in adsorption buffer (AB; 50 mM KCl, 1 mM KPO_4_, 1 mM CaCl_2_, 0.1 mM MgCl_2_, pH 6.5), and supplemented with the protease inhibitor phenylmethylsulfonyl-fluoride (PMSF) at a final concentration of 1 mmol/L. The glass slides were placed as much vertically as possible in 12-well plates and inoculated with* S. mutans* suspension in buffered ultrafiltered UFTYE medium supplemented with 1% (w/v) sucrose and incubated at 37°C, 5% CO_2_.* S. mutans* biofilms were grown undisturbed during 19 h, and later the culture medium was replaced daily until the end of the experimental period, which totalized 72 h (adapted from da Cunha et al. [14]). To assess the effect of the bioactive fractions on* S. mutans* biofilm formation, the 19-hour-old biofilms were treated for two days (10 a.m. and 4 p.m., total of 4 treatments with 1 minute exposure* per* treatment) with the bioactive fractions or vehicle (propylene glycol) at the given concentrations (Table 2), both diluted in sterile AB buffer. On the third day (72 h), the samples were prepared for confocal laser scanning analysis.

For preparation of* C. albicans *biofilms, glass slides (324 mm^2^ surface area) were initially immersed in 12-well flat-bottom cell culture plates (TPP, Trasadingen, Switzerland) containing fetal bovine serum (FBS) (Vitrocell Embriolife, Campinas, SP, Brazil) and incubated aerobically for 24 h at 37°C (pretreatment phase). The slides were then washed with PBS to remove residual FBS, moved to new plates containing a standardized* C. albicans *suspension (5 × 10^6^ CFU/mL) and incubated aerobically for 90 minutes at 37°C (adhesion phase). The slides were gently transferred to new plates to ensure the removal of nonadhered yeasts and were finally immersed in YNB medium containing the EO (Table 2), vehicle (propylene glycol), and standard antifungal (nystatin—7.8 *μ*g/mL (MIC); Sigma-Aldrich, St. Louis, MO, USA), in triplicate. The plates were incubated aerobically at 37°C for 48 h (biofilm formation phase) (adapted from Kuhn et al. [15]).

The sample concentrations used in this study were based on our previous findings of antimicrobial susceptibility for the bioactive fractions and EO [9, 11]. Samples with planktonic minimum inhibitory concentration (MIC) values ranging from 7.8 to 15.6 *μ*g/mL or 15.7 to 125 *μ*g/mL were tested at 600 *μ*g/mL and 800 *μ*g/mL, respectively, in view of the higher microbial resistance observed in biofilm cultures [16].

### 2.6. Confocal Laser Scanning Microscopy (CLSM) Analysis for* S. mutans* Biofilms

Extracellular polysaccharides (EPS) were labeled via incorporation of Alexa Fluor 647 dextran conjugate (D22914, Life Technologies, Carlsbad, CA, USA) (absorbance/fluorescence emission maxima of 647/668 nm), while bacterial cells were stained with SYTO 9 (485/498 nm) (S34854, Life Technologies, Carlsbad, CA, USA) 30 minutes before confocal imaging. The analysis of intact biofilms was performed using a Zeiss LSM 780-NLO confocal laser scanning microscope (Carl Zeiss AG, Germany) equipped with a EC Plan-Neofluar 63x oil immersion objective lens (excitation wavelength 810 nm). Each biofilm was scanned at least at 5 randomly selected points and a confocal image series was generated by optical sectioning (4 *μ*m intervals) at each of the positions [17, 18]. The confocal images stacks were then analyzed with COMSTAT computer program [19] in order to quantify and characterize the 3D structure of the biofilms. The architectural parameters investigated were biovolume (*μ*m^3^·*μ*m^−2^), average thickness (*μ*m), and biofilm coverage (*μ*m) on the slide surface.

### 2.7. CLSM Analysis for* C. albicans* Biofilms

Following formation and treatment of* C. albicans* biofilms, the slides were removed and transferred to a new 12-well plate. Four microliters of FUN-1 (from a 10 mM stock) (F-7030, Life Technologies, Carlsbad, CA, USA) and 15 *μ*L of Concanavalin A, Alexa Fluor 488 Conjugate (from a 5-mg/mL stock) (C11252, Life Technologies, Carlsbad, CA, USA) were mixed into 3 mL of PBS to give final concentrations of 10 *μ*M and 25 *μ*g/mL, respectively, in the wells [15]. The plates were then incubated for 45 min at 37°C and the slides were subsequently analyzed on the confocal microscope equipped with a LD Plan-Neofluar 40x/0.6 water immersion objective lens. Each biofilm was scanned at least at 5 randomly selected points and representative images were selected for each group.

### 2.8. Statistical Analysis

The data were analyzed qualitatively based on the morphology and architecture of EPS and bacterial cells and quantitatively by one-way analysis of variance (ANOVA) followed by Dunnett's multiple comparison test (GraphPad Prism version 5.0, San Diego, California, USA) with type I error set as 0.05. 2D and 3D images were generated and processed using ZEN 2012 Black Edition ©Carl Zeiss Microscopy Gmbh platform. All experiments were performed in triplicate.

## 3. Results and Discussion

### 3.1. Chemical Characterization of Oils and Bioactive Fractions Constituents

The medicinal aromatic plants were obtained exclusively from a germoplasm bank (Table 1) and submitted to chemical monitoring by GC-MS (Table 3), in order to provide georeferencing and traceability.

The chemical composition of the EO and selected bioactive fractions can be seen in Table 3. Importantly, the chemical profile of each EO or active fraction is the reference for each of the studied agents. The phytochemical analysis indicated the presence of volatile compounds, mainly oxygenated mono- and sesquiterpenes, in addition to sesquiterpene hydrocarbons. The major compounds identified in each selected bioactive fraction and EO were guaiol in Ag_4_,* trans*-nerolidol in Bd_2_, thymol in Ls_3_, 1-decanol in Cs_4_, decanal in* C. sativum* EO, and *α*-pinene in* C. articulatus* EO. These compounds alone were proven to have antimicrobial activity against gram-positive bacteria [20] and yeasts [12, 20] that may affect the viability and/or matrix of biofilm.

The fractionation process adopted in this bioguided study is well established in the literature [21, 22]. As the major compounds identified are in accordance with other reports [9, 12], we considered the fractionation process successful.

### 3.2. CLSM Analysis for* S. mutans* Biofilms

Confocal laser scanning microscopy is a useful tool for studying morphology of bacterial biofilms [23], as it allows 3D visualization of hydrated and undisturbed biofilms [24].

**Figure 1:**
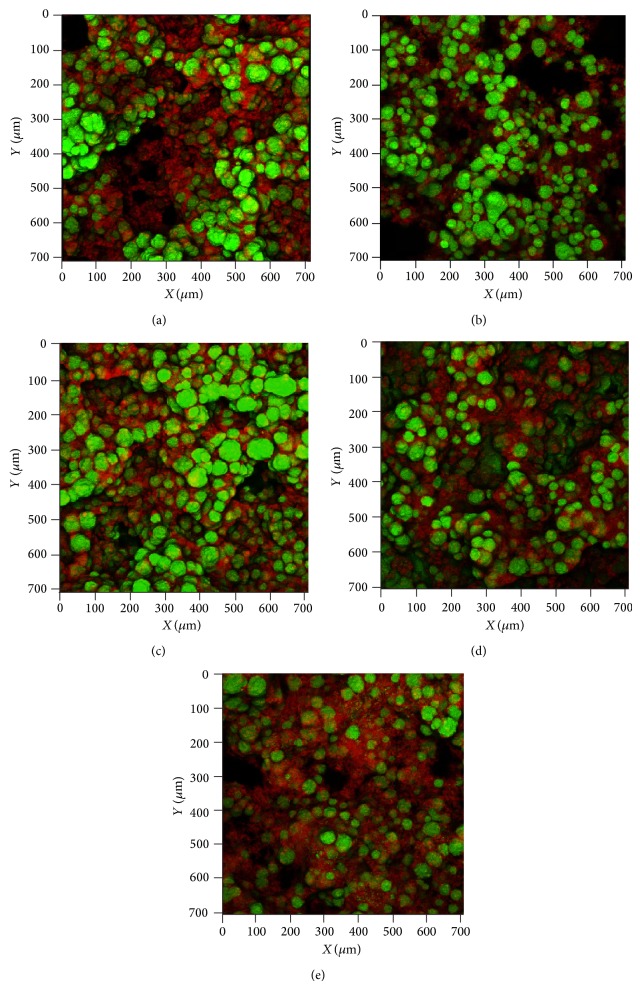
2D confocal imaging: a qualitative analysis. Confocal image stacks of 72-h* S. mutans* UA 159 biofilms following topical treatment with (a)* A. gratissima*: fraction Ag_4_; (b)* B. dracunculifolia*: fraction Bd_2_; (c)* C. sativum*: fraction Cs_4_; (d)* L. sidoides*: fraction Ls_3_; and (e) vehicle (propylene glycol, 6.25% v/v). The structures depicted in red (Dextran, Alexa Fluor 6) represent the extracellular polysaccharides that constitute the biofilm matrix, while the structures depicted in green (Syto 9) are metabolically active bacterial cells (optical magnitude 63x). It can be noted that all bioactive fractions ((a)–(d)) affected the EPS matrix making it less intimately interspersed between and over the cells than did the vehicle alone (e).

**Figure 2:**
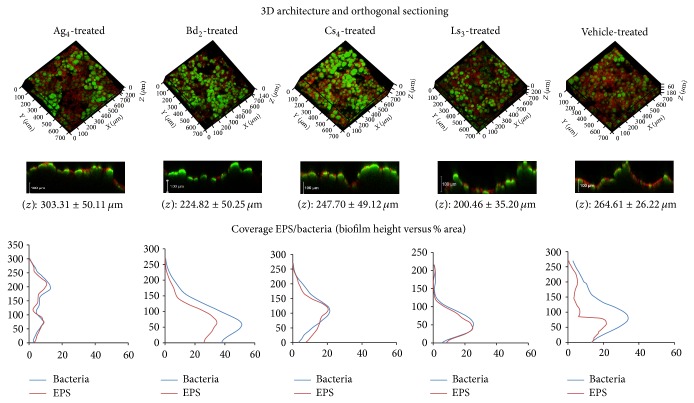
3D confocal imaging: a quantitative analysis. 3D reconstruction of confocal image stacks of 72-h* S. mutans* UA 159 biofilms following topical treatment with* A. gratissima*: fraction Ag_4_;* B. dracunculifolia*: fraction Bd_2_;* C. sativum*: fraction Cs_4_;* L. sidoides*: fraction Ls_3_; and vehicle (propylene glycol, 6.25% v/v). The structures depicted in red (Dextran, Alexa Fluor 6) represent the extracellular polysaccharides that constitute the biofilm matrix, while the structures depicted in green (Syto 9) are metabolically active bacterial cells (optical magnitude 63x). The mean (±SD) of biofilm thickness (*z*) in each group is indicated below the orthogonal images. There were no statistically significant differences in thickness between the groups and the vehicle (*P* > 0.05, One-way ANOVA with Dunnett's posttest). Our coverage (EPS/bacteria) data demonstrate that in all groups the exopolysaccharide matrix was found interspersed between the bacterial cells. Coverage percent represents the percentage of area occupied by bacteria or EPS in each of the CLSM optical section [30].

To the best of our knowledge, this is the first confocal study in the literature determining the effect of these bioactive fractions from EO on the biovolume and architecture of* S. mutans* biofilms. Overall, confocal imaging revealed that most experimental samples at the given concentrations produced both qualitative (Figure 1) and quantitative (Table 4) alterations in the biofilms. However, the analysis of the intacttreated biofilms showed no statistical difference for thickness in all groups compared to the control (vehicle) (*P* > 0.05), as seen in Figure 2.

Table 4 brings the mean (±SD) values of biovolume of 72 h* S. mutans* biofilms treated with the fractions. The biovolume provides an estimation of biofilm biomass in relation to the substratum area [19]. In our study, a significant reduction of both EPS and bacterial cells was observed for Ag_4_ and Ls_3_ (*P* < 0.05) according to the COMSTAT analysis. The groups Bd_2_ and Cs_4_ showed reduced levels of bacterial volume but not of EPS. Therefore, our findings support the view that the bioactive fractions of Ag_4_ and Ls_3_ disrupt biofilm integrity and may have created porosity in the biofilm, as there was no change in thickness but biomass of EPS and bacteria was significantly reduced. This phenomenon could potentially result in a weakened structure and affect the biofilm dynamics, and this hypothesis may be a platform for the development of new antimicrobial targets on biofilm-dependent oral diseases such as caries, candidiasis, and corelated ailments.

An association has been established between biofilm formation and intrinsic resistance to antimicrobial stress [25], which encourages the search for new active molecules with the ability of disrupting biofilm structure. The harmful effects of the fractions upon biofilm assembly that we observed in our study could make the biofilm more susceptible to the action of antimicrobial agents, considering the ease of penetration and access to the cells of the basal layers. With this perspective, synergism studies should be carried out to investigate the combination of topical antibacterial agents, for example, fluoride [21], with these fractions in order to enhance the antimicrobial competence of both products.

As EO are composed of numerous chemical compounds, their antimicrobial activity might be attributed to several distinct mechanisms [8]. Briefly, we can point out that* A. gratissima *and* L. sidoides *fractions had significant effects on bacterial viability acting as bactericides [9] and also affected a key feature of* S. mutans* pathogenicity, that is, production of EPS [3].

Due to their hydrophobicity, EO molecules are able to pass through the cell wall and penetrate fatty acid chains of the phospholipid bilayer, rendering the cell membrane more permeable and causing leakage of intracellular contents. Such loss of cell homeostasis can culminate in lysis and cell death [8]. This mechanism of action may justify the significant decrease in biofilm cell population caused by the fractions in relation to the vehicle.

EPS are an outstanding key-factor in biofilm formation being produced by glycosyltransferases [26]. The fractions from* A. gratissima *and* L. sidoides* reduced its amount, indicating that one of their putative mechanisms of action might be related to the inhibition of glycosyltransferase activity.

Our coverage (EPS/bacteria) data (Figure 2) demonstrate that, in all groups, the EPS matrix was found interspersed between the bacterial cells. Biofilm distribution was assessed by measuring the percentage of coverage of the substrate by EPS and bacteria [19].

### 3.3. CLSM Analysis for* C. albicans* Biofilms

In previous studies,* C. sativum* [11] and* C. articulatus* [10] have stood out for their promising anti-*Candida* activity. We reported that* C. sativum* EO, rich in decanal and* trans*-2-decenal (major compounds), has strong fungicidal effects against* Candida albicans* and non-*albicans* and acts by binding to membrane ergosterol, which increases ionic permeability and leads to cell death. This is the same mechanism of action of polyenes such as nystatin and amphotericin B [27].* C. articulatus *presents antifungal properties due to constituents such as *α*-pinene (major compound) that could be able to destroy cell integrity and inhibit respiration and the ion transport processes, leading to cell death [28].

Both* C. sativum* and* C. articulatus* EO inhibited yeast biofilm adherence onto a polystyrene substrate from 62.5 *μ*g/mL [11] and 1.95 *μ*g/mL [10], respectively. It has also been found that the EO from these species are more effective against* Candida* spp. than their fractions either on planktonic or biofilm cultures [9, 11, 12]; that is why we decided to carry out the tests using only the EO, taking into account costs of fractionation and biological activity.

**Figure 3:**
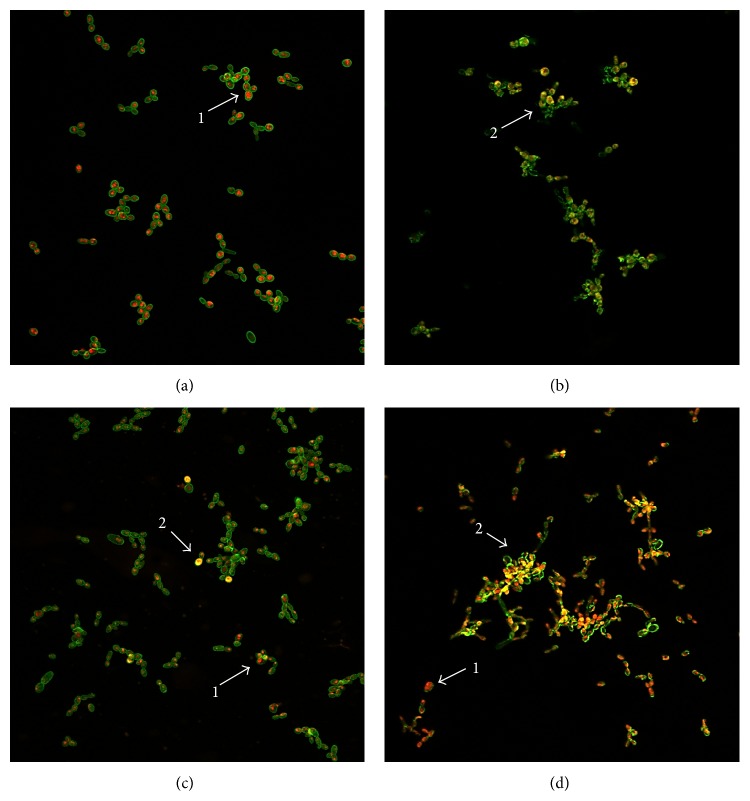
Inhibitory effects on* Candida* biofilm. 2D confocal imaging of* C. albicans* CBS 562 biofilm treated with (a) vehicle (propylene glycol, 6.25% v/v); (b) standard antifungal (nystatin); (c)* C. articulatus* crude oil; and (d)* C. sativum* crude oil. The structures depicted in green (Concanavalin A, Alexa Fluor 488 Conjugate) represent the yeast cell wall and those depicted in yellow (FUN 1 Cell Stain) are nonviable cells, metabolically inactive (arrow 2). The viable cells, in turn, convert the dye FUN-1 to red fluorescent aggregates (arrow 1) (40x optical magnitude). Concanavalin A selectively binds to polysaccharides, including alpha-mannopyranosyl and alpha-glucopyranosyl residues, and gives a green fluorescence. FUN-1 is a fluorescent dye taken up by yeast cells; in the presence of metabolic viability it is converted from a diffuse yellow cytoplasmic stain to red [15]. It can be noted that* C. sativum* essential oil drastically affected the viability of* C. albicans* cells when compared to the vehicle and standard antifungal.

In our study, confocal microscopy revealed that both OE altered the viability of yeast cells compared to the vehicle (Figure 3), most notably* C. sativum* EO (Figure 3(d)), which substantially decreased the metabolic activity of the fungal cells. Based on these and other results,* C. sativum* EO emerges as a promising candidate for nonclinical and clinical toxicology testing for the development of new drugs to treat denture-related oral candidiasis.

The treatment with* C. sativum* EO (Figure 3(d)) affected the viability of yeasts as much as did the standard antifungal currently employed in dental practice (Figure 3(b)). This finding is interesting if one compares an EO (complex mixture of chemical compounds) classified as food in Brazil and worldwide as GRAS (generally regarded as safe) [29] with a synthetic monodrug (nystatin), and this may justify the ethnopharmacology of the popular use of coriander as a rinse to control denture-related atrophic candidiasis (folk medicine).

## 4. Conclusion

The bioactive fractions from* A. gratissima *and* L. sidoides* significantly reduced the biovolume of extracellular polysaccharides and bacterial cells in the* S. mutans* biofilm model used, but there was no difference with regard to biofilm thickness. Taken together, our findings support the view that these fractions disrupt biofilm integrity and may have created porosity in the biofilm, as biomass decreased but thickness was unaltered. Furthermore,* C. sativum* EO drastically affected the viability of* C. albicans* cells. These results highlight the promising antimicrobial activity of these plant species and suggest avenues for future translational research on the treatment of dental caries and oral candidiasis.

## Figures and Tables

**Table 1:** Ethnobotanical characterization of the plant species used in this study.

Family	Botanical name	Source	CPMA registration number^*^	Folk name
Verbenaceae	*Aloysia gratissima *	leaf	714	Brazilian lavender
Asteraceae	*Baccharis dracunculifolia *DC	leaf	1841	Broom weed
Apiaceae	*Coriandrum sativum* L.	leaf	664	Coriander
Cyperaceae	*Cyperus articulatus *Vahl.	bulbs	222	Priprioca
Verbenaceae	*Lippia sidoides *Cham.	leaf	398/399	Rosemary

^*^Website: http://webdrm.cpqba.unicamp.br/cpma/banco_de_dados/index.php?centro=catalogo.

**Table 2:** Concentration of the bioactive fractions and crude oils tested in this bioguided study against *S. mutans* and *C. albicans*, respectively.

Plant species	Sample	Microorganism	Concentration
*A. gratissima *	Fraction Ag_4_	*S. mutans* UA 159	800 *µ*g/mL
*B. dracunculifolia *	Fraction Bd_2_	*S. mutans* UA 159	600 *µ*g/mL
*L. sidoides *	Fraction Ls_3_	*S. mutans* UA 159	800 *µ*g/mL
*C. sativum *	Fraction Cs_4_	*S. mutans* UA 159	600 *µ*g/mL
*C. sativum *	Crude oil	*C. albicans *CBS* 562 *	600 *µ*g/mL
*C. articulatus *	Crude oil	*C. albicans *CBS* 562 *	800 *µ*g/mL

**Table 3:** Major chemical compounds of the selected bioactive fractions and EO identified by GC-MS.

Rt (min)^a^	RI^b^	Major compounds^*^	Relative percentage^c^					
			Ag_4_	Bd_2_	Ls_3_	Cs_4_	CS	CA
4.82	933	*α*-Pinene						7.82
13.06	1140	*trans*-Pinocarveol	13.16					6.02
15.41	1197	Myrtenol	5.31					
15.38	1207	Decanal					19.09	
17.74	1263	*trans*-2-Decenal					17.54	
18.59	1271	1-Decanol				16.93		
18.15	1272	2-Decen-1-ol					12.33	
18.26	1275	Cyclodecane					12.15	
19.43	1291	Thymol			93.98	5.17		
21.84	1349	Ethyl ester benzenepropanoic		11.7				
20.72	1373	*α*-Copaene						6.79
24.77	1419	*trans*-Caryophyllene		10.7		9.45		
26.19	1467	*cis*-2-Dodecenal					10.72	
26.83	1470	*trans*-2-Dodecen-1-ol				5.75		
26.05	1503	*α*-Bulnesene						6.87
30.59	1566	*trans*-Nerolidol		52.2				
28.69	1572	M^d^ = 220						6.10
31.02	1577	Spathulenol		11.5		12.39		
31.23	1582	Caryophyllene oxide		6.3				
31.26	1583	Globulol				12.66		
31.92	1601	Guaiol	29.63					
33.89	1654	*α*-Cadinol				5.43		
34.41	1669	Bulnesol	11.79					
34.02	1671	M = 210					11.51	
32.52	1675	Mustakone						6.06
35.06	1747	M = 218						6.48

Notes: ^a^retention time; ^b^retention index; ^c^percentage fraction of the total area integrated for the chromatogram; ^d^M: molecular weight of a nonidentified compound. ^*^Only the compounds with relative percentage above 5% are listed. Ag_4_: *A. gratissima* fraction 4; Bd_2_: *B. dracunculifolia* fraction 2; Ls_3_: *L. sidoides* fraction 3; Cs_4_: *C. sativum* fraction 4; CS: *C. sativum* crude oil; CA: *C. articulatus* crude oil.

**Table 4:** Mean values (±SD) of bacterial cells and extracellular polysaccharides biomass, expressed in *μ*m^3^·*μ*m^−2^, of topically treated biofilms of *Streptococcus mutans* UA 159.

Group	*S. mutans* cells	Exopolysaccharide matrix (EPS)
*A. gratissima *(Ag_4_)	15.63 ± 2.56▼	14.90 ± 7.73▼
*B. dracunculifolia *(Bd_2_)	33.41 ± 19.16▼	27.99 ± 14.85▲
*C. sativum *(Cs_4_)	22.83 ± 12.63▼	20.49 ± 12.08▲
*L. sidoides *(Ls_3_)	17.45 ± 4.34▼	16.68 ± 7.01▼
Vehicle	36.32 ± 32.77	20.00 ± 3.41

Note: the arrow “▼” indicates statistically significant reduction of cells or EPS compared to the control, whereas the arrow “▲” indicates statistically significant increase of cells or EPS compared to the control (one-way ANOVA with Dunnett's multiple comparison test, with a significance level of 5%).
